# A novel algorithm for cardiovascular screening using conjunctival microcirculatory parameters and blood biomarkers

**DOI:** 10.1038/s41598-022-10491-7

**Published:** 2022-04-21

**Authors:** Agnes Awuah, Julie S. Moore, M. Andrew Nesbit, Mark W. Ruddock, Paul F. Brennan, Jonathan A. Mailey, Andrew J. McNeil, Min Jing, Dewar D. Finlay, Emanuele Trucco, Mary Jo Kurth, Joanne Watt, John V. Lamont, Peter Fitzgerald, Mark S. Spence, James A. D. McLaughlin, Tara C. B. Moore

**Affiliations:** 1 Biomedical Sciences Research Institute, Ulster University, Cromore Road, Coleraine, BT52 1SA UK; 2 Clinical Studies Group, Randox Laboratories Ltd, 55 Diamond Road, Crumlin, BT29 4QY UK; 3 Department of Cardiology, Royal Victoria Hospital, Belfast Health and Social Care Trust, 274 Grosvenor Road, Belfast, BT12 6BA UK; 4 VAMPIRE Project, Computing (SSEN), University of Dundee, Dundee, DD1 4HN UK; 5 Nanotechnology and Integrated Bioengineering Centre (NIBEC), Ulster University, Jordanstown, BT37 0QB UK

**Keywords:** Biomarkers, Cardiology, Health care, Medical research

## Abstract

Microvascular haemodynamic alterations are associated with coronary artery disease (CAD). The conjunctival microcirculation can easily be assessed non-invasively. However, the microcirculation of the conjunctiva has not been previously explored in clinical algorithms aimed at identifying patients with CAD. This case–control study involved 66 patients with post-myocardial infarction and 66 gender-matched healthy controls. Haemodynamic properties of the conjunctival microcirculation were assessed with a validated iPhone and slit lamp-based imaging tool. Haemodynamic properties were extracted with semi-automated software and compared between groups. Biomarkers implicated in the development of CAD were assessed in combination with conjunctival microcirculatory parameters. The conjunctival blood vessel parameters and biomarkers were used to derive an algorithm to aid in the screening of patients for CAD. Conjunctival blood velocity measured in combination with the blood biomarkers (N-terminal pro-brain natriuretic peptide and adiponectin) had an area under receiver operator characteristic curve (AUROC) of 0.967, sensitivity 93.0%, specificity 91.5% for CAD. This study demonstrated that the novel algorithm which included a combination of conjunctival blood vessel haemodynamic properties, and blood-based biomarkers could be used as a potential screening tool for CAD and should be validated for potential utility in asymptomatic individuals.

## Introduction

Cardiovascular disease (CVD) is a major cause of morbidity and mortality in developed countries^1^. It accounts for 17.9 million deaths globally, with up to 80% of these deaths resulting from myocardial infarction (MI) and stroke^1^. Coronary artery disease (CAD) is the most common type of CVD and results from the accumulation of lipid deposits (atherosclerosis) in the coronary arteries^2^. CAD is the leading cause of death in developed countries^3^.

CVD not only carries a significant morbidity and mortality burden but has major economic implications. The World Heart Foundation estimated the total cost of CVD was US$863 billion in 2010, with a predicted increase to approximately US$1,044 billion by 2030^4^. The total expenditure on CVD in the EU is €210 billion annually with 53% (€111 billion) due to direct health care expenditures, productivity losses cost 26% (€54 billion), and informal care of patients costs 21% (€45 billion)^5^.

Atherosclerosis is the key pathophysiological process that underlies CAD^6^. This process develops over a prolonged period of time and is influenced by a variety of medical and behavioural risk factors^5^. Atherosclerotic plaques in general do not produce symptoms until the affected vessel has significant luminal narrowing and the resulting ischaemic cascade can be silent in its early stages. Prior to atherosclerotic plaque rupture and resultant MI, patients may be asymptomatic. Many predictive approaches, screening and diagnostic methods for atherosclerotic diseases have evolved. These include utilization of independent traditional and biochemical risk factors, general CVD risk scores, and assessment of cardiac microvasculature^6–11^.

Current European Society of Cardiology (ESC) clinical guidelines recommend the systematic assessment of cardiovascular (CV) risk in men > 40 years and in women > 50 years or post-menopausal with no CV risk factors^10^. ESC assessment of risk for asymptomatic individuals utilises the Systematic Coronary Risk Estimation (SCORE) assessment tool, whilst National Institute for Clinical Excellence (NICE) advocates the use of QRISK3^12,13^. Both tools take into consideration conventional vascular risk factors to estimate long-term CV risk. The results of these estimates of risk are then used to inform GPs’ decisions with regards to primary preventative therapies and overall long-term risk. The risk score obtained may be modified by the presence of pre-existing medical conditions that were not taken into consideration during the initial risk calculation (e.g. chronic kidney disease or diabetes mellitus); or with the detection of preclinical asymptomatic vascular damage using imaging modalities (e.g. computed tomography (CT) coronary calcium score, carotid ultrasound or ankle brachial pressure index^13^). All pre-existing screening methods are limited either by the exposure of the patient to ionizing radiation (CT); or by the need for expensive imaging modalities requiring operator expertise to perform and interpret (carotid ultrasound)^11,14^. Due to the global and regional health and socioeconomic implications of CAD, there is a need to identify novel factors for screening asymptomatic individuals for early detection of CAD.

Previously heart-type fatty acid-binding protein (H-FABP), for example, has been shown to be an early blood diagnostic biomarker of MI^15^. H-FABP is detected in blood within 30 min of an ischaemic event. Measurement of H-FABP with troponin proved to be a reliable diagnostic tool for the early diagnosis of MI and a valuable rule out test at presentation to the emergency department^15,16^. Cholesterol is usually measured in screening > 40-year-old males and > 50-year-old females. Measurement of biomarkers in addition to cholesterol may provide further information to identify individuals at risk of CAD in asymptomatic individuals as a screening tool.

Microcirculatory studies have demonstrated the association of inflammation with atherosclerosis^17–20^. In addition, it has been shown that endothelial dysfunction in the microcirculation is an early manifestation and marker of vascular disease^21^. Microcirculatory changes have been observed in patients with hypertension, diabetes, sickle cell disease and sepsis^17,21–23^. A change in microcirculation is a potential marker for determining risk of potential major adverse cardiovascular events (MACE). These changes may manifest in changes in blood biomarker levels associated with these processes [e.g. NT-proBNP, interleukin-6 (IL-6), C-reactive protein (CRP), H-FABP, high density lipoprotein (HDL), fibrinogen, apolipoproteins, adiponectin, HDL-3 and monocyte chemoattractant protein-1 (MCP-1)].

In addition, the networks of the human eye allow examination of the microcirculation at the anterior (ocular adnexum) and posterior (retina) aspect that can easily be visualised and accessed non-invasively^24^. Changes to the retinal vessel diameters show evidence of diseases that are conventional risk factors for CAD (e.g. diabetes and hypertension)^25–27^ and hence could play a pathophysiological role in CVD risk stratification and prediction^28^. Blood flow within the conjunctival vasculature can be directly visualised and hence assessment of microcirculatory parameters (e.g. velocity (V), flow (Q), wall shear rate (WSR)) can also be measured non-invasively^29–31^. The study by Brennan et al.^31^ assessed groups of subjects with and without hypertension, diabetes mellitus, dyslipidaemia, reduced left ventricular ejection fraction (< 50%), as well as smokers vs. non-smokers. The results show only the blood flow rate (pl/s) increased significantly in diabetics vs. non-diabetics respectively (181 ± 61 vs. 151 ± 39 pl/s, p = 0.04). Previous studies have identified conjunctival microvascular changes in diseases such as sickle cell, hypertension and diabetes mellitus^32,33^.

The aim of this study was to develop a screening tool that could be used to identify patients at risk of CAD. Our previous study detected changes in conjunctival microvascular measurements between control and post-MI patients^31^. As part of the study, blood samples were collected to determine if blood biomarkers could be used in conjunction with microvascular measurements to improve the diagnostic potential of the test.

## Methods

### Study population

In this case–control MACE study, we compared a cohort of inpatients with severe cardiovascular phenotype after an acute MI with a healthy gender-matched patient cohort. Participants < 18 years were excluded from the study. All study participants were recruited between January 2018 and November 2019. Participants were eligible for inclusion in the ‘healthy’ cohort if they had no previous history of CAD.

Patients were eligible for inclusion in the post-MI cohort if they had been admitted to hospital with a diagnosis of MI that fulfilled the European Society of Cardiology (ESC) 4th Universal definition of type 1 MI. This is defined as acute myocardial injury with clinical evidence of acute myocardial ischaemia. A fall and/or rise of cardiac troponin (cTn) with 1 or more values beyond the 99th percentile of the Upper Reference Limit (URL), in addition to one or more of the following: evidence of new ischaemic ECG changes; pathological Q waves; current loss of myocardium or regional wall motion abnormality in line with an ischaemic aetiology; and coronary thrombus^34^.

The study complied with ethical principles on human experimentation (Declaration of Helsinki) and was approved by the office for Research Ethics Committee Northern Ireland (ORECNI) (IRAS Reference-166742), and Research Governance Committee of Belfast Trust (Trust Reference-15144TM-AS). Written informed consent was obtained from each participant prior to recruitment. The study was conducted in accordance with Standard for Reporting Diagnostic Accuracy (STARD) guidelines^35^.

### Clinical data collection

All participants were asked to complete a clinical questionnaire detailing baseline demographics, past medical history, current medications, family history of medical conditions and lifestyle information. Electronic healthcare records were reviewed following ethical approval and participants’ consent for medical history to ensure accuracy. Baseline measurement of blood pressure, heart rate, oxygen saturations, height, and weight were also performed.

### Blood sampling and laboratory methods

For biomarker assessment, 24mls of venous blood was sampled. Serum and plasma samples were analysed by Randox Clinical Laboratory Services (RCLS), (Antrim, UK) on cytokine arrays (Randox Laboratories Ltd, Crumlin, UK); using an Evidence Investigator analyser (Randox Laboratories Ltd, Crumlin, UK) for the following proteins: Cytokine array 1: Interleukin (IL)-1α, -1β, -2, -4, -6, -8, -10, vascular endothelial growth factor (VEGF), epidermal growth factor (EGF), tumour necrosis factor alpha (TNF-α), interferon gamma (IFNɣ) and MCP-1. H-FABP, adiponectin, homocysteine and HDL-3 were measured on the RX Imola analyser (Randox, Crumlin, UK). Folate and vitamin B_12_ were measured on a Cobas8000 (Roche, Basel, Switzerland).

Asymmetric dimethylarginine (ADMA) and leucine-rich alpha-2-glycoprotein-1 (LRG-1) were assessed by ELISA, according to manufacturer’s instructions (ELISAgenie, Ireland). The limits of detection (LOD) for the biomarkers were as follows: IL-1α 0.19 pg/ml; IL-1β 0.26 pg/ml; IL-2 2.97 pg/ml; IL-4 2.12 pg/ml; IL-6 0.12 pg/ml; IL-8 0.36 pg/ml; IL-10 0.37 pg/ml, VEGF 3.24 pg/ml; EGF 1.04 pg/ml; TNF-α 0.59 pg/ml; IFNɣ 0.44 pg/ml; MCP-1 3.53 pg/ml; H-FABP < 2.94 ng/ml; adiponectin 0.18 µg/ml; folate 2.72 nmol/l; homocysteine 1.74 µmol/l; vitamin B_12_ 73.8 pmol/l; HDL-3 4 mg/dl; plasma ADMA < 0.938 µmol/L; plasma LRG-1 < 0.0047 µg/ml. Results below the LOD were inputted as 90% of the LOD^36^.

Troponin, lipids, NT-proBNP, urate, urea and electrolytes and all other biochemical measurements, were analysed by clinical staff at the Kelvin Building Laboratories, Royal Victoria Hospital, Belfast. Apo-lipoprotein A and B were analysed by the Biochemistry Department, Cardiff and Vale University Hospital, UK as reported previously^31^.

### Conjunctival vessel imaging

**Figure 1:**
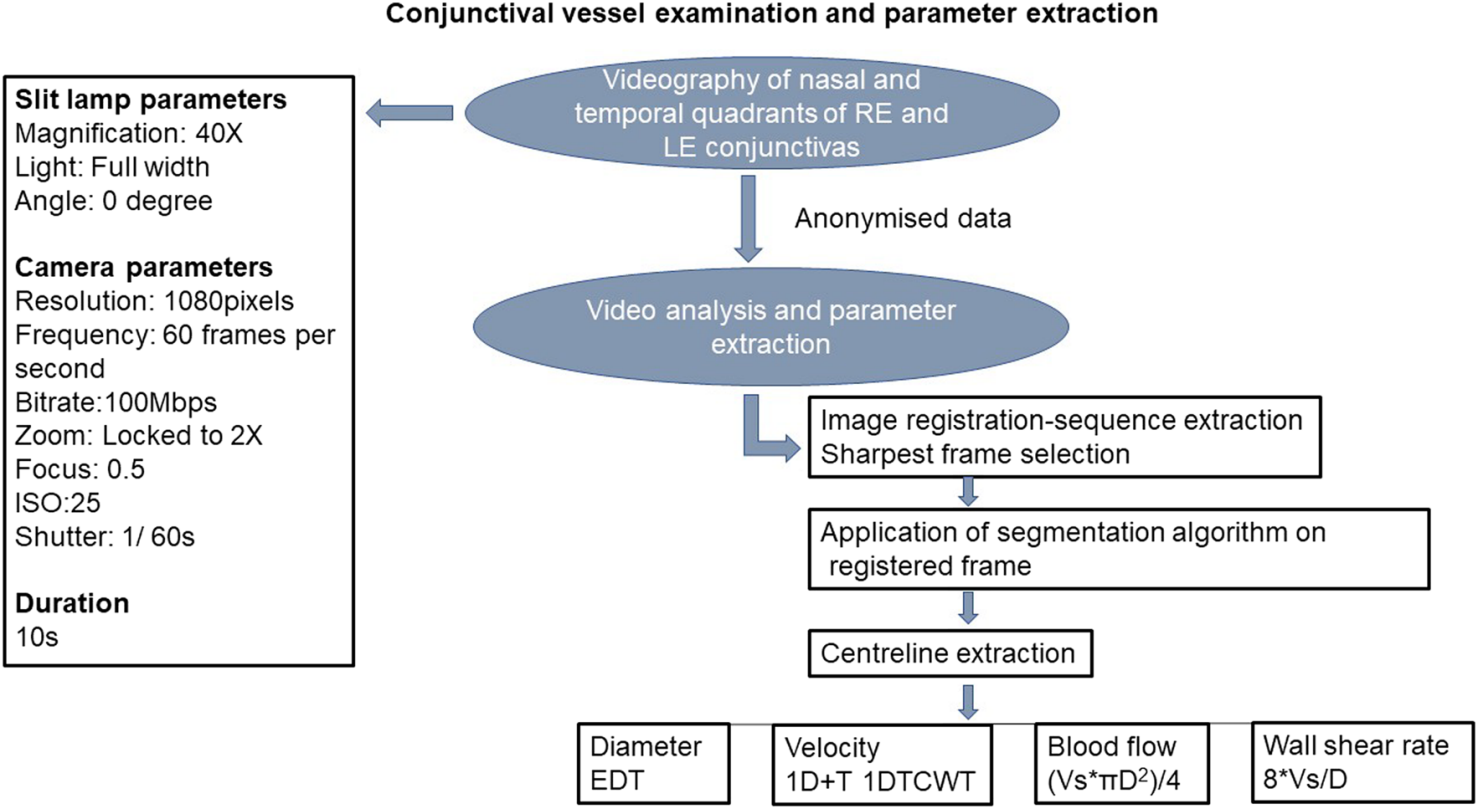
Vessel parameter data collection. *RE* Right Eye, *LE* Left Eye, *EDT* Euclidean Distance Transform, *1D + T* 1 Dimension + Time, *CWT* Continuous Wavelet Transform, *Vs* Cross Sectional Velocity, *D* Diameter.

Conjunctival microvascular imaging was performed using our previously described non-invasive imaging tool (iPhone 6s with slit lamp biomicroscope) and semi-automated software; developed by the VAMPIRE centre (Dundee) and NIBEC (Ulster University) for quantifying vessel haemodynamic properties (vessel diameter (D), axial velocity (Va), cross-sectional velocity (Vs), Q, and WSR)^30,37,38^. The process is summarised as a flow diagram in Fig. 1.

Briefly, the imaging tool consisted of a 2X magnification device (Apple iPhone 6s) adapted as an eye piece of a slit-lamp biomicroscope (Topcon SL-D4) with a 40 × magnification lens. A third-party camera application (ProMovie Recorder) was used for controlling camera settings (focus, shutter speed and ISO). Conjunctival imaging may take around 5 to 10 min per patient. Videos were obtained from conjunctival vessels in the nasal and temporal bulbar conjunctivas while the participants focused on a fixation target attached to the slit lamp. A total of 4 videos per participant were obtained in 40 s; each video was captured at 60 frames per second for 10 s. While all videos were captured under the same, or similar, lighting conditions in a hospital setting, the brightness of the light from the slit-lamp source rendered any small fluctuations in ambient lighting insignificant. Anonymised videos taken during the eye examinations were processed and analysed by researchers blinded to the participant baseline demographics and cohort allocation at Dundee University and Ulster University. They were analysed with set-up specific semi-automated software^38^. Measurements from vessels of both eyes were combined for the estimation of overall mean of each parameter per patient (patient’s mean).

### Estimation of conjunctival microcirculatory parameters

Estimation of blood vessel parameters has been previously described^30,37–39^. Video sequences with minimal motion artefact were selected. The sharpest frame in the sequence was selected as a reference frame and all other frames registered to it. A segmentation algorithm was applied to segment vessels before vessel centrelines were extracted for estimating the dynamic properties of the vessels. Euclidean Distance Transformation was used for estimation of vessel diameter. The axial velocity (Va) was estimated based on spatial–temporal image (STI) via applying one dimension of space plus time (1D + T) continuous wavelet transform (1DTCWT)^40^. Blood flow was calculated from the product of the cross-sectional velocity (Vs) and diameter (D), using the formula, (Vs.πD^2^)/4. Wall shear rate was calculated by the formula 8Vs/D^30,37,38^.

### Statistical analysis

Statistical analyses were performed using IBM SPSS v25 and R version 4.1.2^41^. The following statistics were analysed on the appropriate data: descriptive statistics (mean ± SD) and percentages for summarizing parametric and nominal data, respectively; Chi square and Pearson’s tests for determining the association and correlation between variables; and independent t-test for differences between group means for parametric data. Where the assumptions of t-test were unmet, the Mann–Whitney *U* test or Wilcoxon rank-sum test was used. Variable medians were compared when distributions were similar but mean ranks were compared when distributions were dissimilar. Binary logistic regression and receiver operator characteristic (ROC) curves were used to test sensitivity and specificity. Normality was assessed by Shapiro Wilk test (p > 0.05). Data transformation was applied when the assumption of normality was unmet. Statistical significance was set at an α-level p < 0.05.

## Results

### Demographic characteristics

A total of n = 132 participants were recruited to the MACE study. There was an equal number of participants in each group; post-MI n = 66 and healthy controls n = 66. Post MI cases were recruited 2.53 ± 3.44 days (range 0–21 days) after hospital admission following type 1 MI. There was a significant difference between the mean ages of the control and MI groups (p = 0.039) but no significant difference between gender distribution (χ^2^(1) = 1.91, p = 0.168). Demographic and clinical characteristics are described in Table 1.

**Table 1:** Demographic and clinical characteristics (mean ± SD).

Variables	Control n = 66	Post-MI n = 66	p value
Age (years)	52.50 ± 9.73	56.73 ± 11.41	0.039
Gender (males)	45/66 (68.2%)	52/66 (78.8%)	0.168
Height (cm)	168.21 ± 10.68	170.77 ± 9.34	0.262
Weight (kg)	81.40 ± 22.72	83.59 ± 14.93	0.186
Body mass index (kg/m^2^)	28.71 ± 7.66	28.62 ± 4.68	0.306
Systolic BP (mmHg)	128 ± 16	120 ± 16	0.006
Diastolic BP (mmHg)	76 ± 10	73 ± 11	0.066
Heart rate (beats/min)	71 ± 10	73 ± 12	0.299
Hypertension	8/66 (12.1%)	31/66 (47.0%)	0.001
Diabetes	2/66 (3.0%)	15/66 (22.7%)	0.001
COPD	6/66 (9.1%)	8/66 (12.1%)	0.572
Hypercholesterolemia	17/64 (26.6%)	36/66 (54.5%)	0.001
Previous MI	0/66 (0%)	9/66 (13.6%)	0.002
Heart failure	0/66 (0%)	6/65 (9.2%)	0.012
Stroke	0/66 (0%)	1/66 (1.5%)	0.315
Smoking (yes)	29/66 (43.9%)	43/66 (65.2%)	0.014
Family history of IHD	21/66 (31.8%)	38/66 (57.6%)	0.003

*COPD* chronic obstructive pulmonary disease, *IHD* ischaemic heart disease, *BP* blood pressure.

### Clinical and lifestyle characteristics

None of the participants in the control group had previous MI (control: 0/66 (0.00%) vs. post-MI: 9/66 (13.6%), heart failure (control: 0/66 (0.00%) vs. post-MI: 6/66 (9.2%) or stroke (control 0/66 (0.00%) vs. post-MI:1/66 (1.5%), p < 0.05 for all variables). There were more smokers in the post-MI group; 43/66 (65.2%) vs. 29/66 (43.9%); p = 0.014, respectively. No significant differences in height were found between the cohorts. The mean and standard deviation values of height (cm) for both cohorts, controls vs. MI respectively were 168.21 ± 10.68 cm vs. 170.77 ± 9.34 cm, p = 0.262.

### Comparison of microvascular parameters and biomarkers

Results of the comparison for microvascular parameters between groups are described in Table 2.

**Table 2:** Ocular microvascular parameters (mean ± SD).

Variables	Control n = 66	Post-MI n = 66	p value
Diameter (D, μm)	21.45 ± 3.03	22.79 ± 3.07	0.012
Axial velocity (Va, mm/s)	0.54 ± 0.05	0.50 ± 0.06	0.001
Cross-sectional velocity (Vs, mm/s)	0.38 ± 0.04	0.35 ± 0.04	0.001
Blood flow (Q, pl/s)	159.66 ± 47.27	161 ± 48.12	0.457
Wall shear rate (WSR, s^−1^)	167.78 ± 34.37	143.59 ± 28.56	0.001

A summary of biomarker results between the control and post-MI group are described in Table 3.

**Table 3:** Biomarker results (mean ± SD).

Variables	Control n = 66	Post MI n = 66	p value
HbA1c (mmol/l)	39.25 ± 8.29	45.27 ± 16.89	0.053
Sodium (mmol/l)	140.28 ± 1.77	138.95 ± 2.52	0.001
Potassium (mmol/l)	4.40 ± 0.31	4.33 ± 0.36	0.401
Urea (mmol/l)	5.38 ± 1.18	5.47 ± 2.18	0.541
Creatinine (µmol/l)	79.28 ± 16.66	84.05 ± 22.21	0.272
Creatinine clearance (ml/min)	110 ± 42.42	104.80 ± 34.93	0.733
HGB (g/l)	146.38 ± 11.00	142.39 ± 15.44	0.095
Haematocrit (l/l)	0.43 ± 0.03	0.42 ± 0.04	0.145
White cell count (10^9^ µl/l)	6.76 ± 1.73	8.90 ± 3.01	0.001
Platelet count (10^9^/l)	263.17 ± 46.54	262.59 ± 68.75	0.426
MCV (fl)	88.93 ± 4.30	88.20 ± 5.93	0.438
CRP (mg/l)	3.24 ± 6.39	20.96 ± 42.49	0.001
NT-proBNP (ng/l)	74.70 ± 186.68	1049.11 ± 1663.65	0.001
Total cholesterol (mmol/l)	5.11 ± 0.89	4.65 ± 1.44	0.005
Triglyceride (mmol/l)	1.67 ± 0.96	1.97 ± 1.35	0.217
HDL (mmol/l)	1.48 ± 0.48	1.13 ± 0.30	0.001
LDL (mmol/l)	2.85 ± 0.72	2.69 ± 1.33	0.124
Non-HDL (mmol/l)	3.62 ± 0.97	3.52 ± 1.41	0.404
Chol-HDL ratio	3.79 ± 1.37	4.31 ± 1.58	0.048
Prothrombin Time (s)	10.52 ± 0.50	10.94 ± 0.70	0.001
APTT (s)	26.47 ± 2.19	35.17 ± 29.28	0.468
Fibrinogen (g/l)	3.12 ± 0.61	3.89 ± 1.05	0.001
Urate (mmol/l)	0.32 ± 0.07	0.35 ± 0.11	0.280
Apolipoprotein A (g/l)	1.54 ± 0.29	1.34 ± 0.25	0.001
Apolipoprotein B (g/l)	1.02 ± 0.23	1.04 ± 0.31	0.949
Folate (nmol/l)	8.23 ± 6.06	6.45 ± 4.15	0.088
Homocysteine (µmol/l)	14.35 ± 6.96	15.81 ± 7.15	0.084
Vitamin B_12_ (pmol/l)	436.97 ± 210.39	396.92 ± 174.68	0.348
Adiponectin (µg/ml)	11.15 ± 6.20	8.58 ± 5.92	0.001
H-FABP (ng/ml)	3.62 ± 2.04	13.75 ± 29.73	0.001
HDL-3 (mg/dl)	20.17 ± 4.51	17.73 ± 4.75	0.001
Serum IL-1α (pg/ml)	0.48 ± 0.13	0.47 ± 0.13	0.320
Serum IL-1β (pg/ml)	2.38 ± 2.77	1.98 ± 0.50	0.550
Serum IL-2 (pg/ml)	4.25 ± 5.62	3.46 ± 1.37	0.673
Serum IL-4 (pg/ml)	2.80 ± 1.77	2.43 ± 0.47	0.332
Serum IL-6 (pg/ml)	1.92 ± 2.36	8.17 ± 16.77	0.001
Serum IL-8 (pg/ml)	10.87 ± 8.40	13.37 ± 13.56	0.082
Serum IL-10 (pg/ml)	1.38 ± 0.71	1.39 ± 0.46	0.469
Serum MCP-1 (pg/ml)	236.54 ± 100.32	201.79 ± 124.48	0.006
Serum TNF-α (pg/ml)	2.96 ± 2.17	2.75 ± 0.87	0.594
Serum VEGF (pg/ml)	120.62 ± 102.64	162.15 ± 123.21	0.043
Serum IFNɣ (pg/ml)	0.69 ± 0.67	0.98 ± 2.91	0.523
Serum EGF (pg/ml)	80.79 ± 41.54	60.89 ± 38.96	0.011
Plasma IL-1α (pg/ml)	0.44 ± 0.14	0.43 ± 0.13	0.796
Plasma 1L-1β (pg/ml)	2.42 ± 4.35	1.83 ± 0.52	0.366
Plasma IL-2 (pg/ml)	4.03 ± 5.23	3.31 ± 1.08	0.982
Plasma IL-4 (pg/ml)	2.82 ± 1.81	2.30 ± 0.49	0.031
Plasma IL-6 (pg/ml)	1.99 ± 3.00	7.06 ± 10.88	0.001
Plasma IL-8 (pg/ml)	4.74 ± 4.04	5.20 ± 8.14	0.564
Plasma IL-10 (pg/ml)	1.29 ± 0.46	1.43 ± 0.52	0.094
Plasma MCP-1 (pg/ml)	90.41 ± 37.16	87.13 ± 70.29	0.103
Plasma TNF-α (pg/ml)	2.95 ± 5.48	2.33 ± 0.87	0.347
Plasma VEGF (pg/ml)	24.92 ± 21.48	20.22 ± 9.97	0.468
Plasma IFNɣ (pg/ml)	0.63 ± 0.57	1.04 ± 2.88	0.478
Plasma EGF (pg/ml)	21.09 ± 16.39	16.04 ± 15.29	0.060
ADMA (µmol/l)	3.15 ± 1.68	3.63 ± 11.93	0.258
LRG-1 (µg/ml)	14.79 ± 9.44	11.93 ± 4.92	0.048

*HbA1c* glycated haemoglobin, *HGB* haemoglobin, *MCV* corpuscular volume, *HDL* high-density lipoprotein, *LDL* low-density lipoprotein, *Chol-HDL* cholesterol-high-density lipoprotein, *NT-proBNP* N-terminal pro brain natriuretic peptide, *H-FABP* heart-type fatty acid-binding protein, *CRP* C-reactive protein, *IL* interleukin, *MCP-1* monocyte chemoattractant protein-1, *VEGF* vascular endothelial growth factor, *TNF-α* tumour necrosis factor alpha, *IFNɣ* interferon gamma, *EGF* endothelial growth factor, *ADMA* asymmetrical dimethylarginine, *LRG-1* leucine-rich alpha-2-glycoprotein-1.

### Ocular-biomarker algorithm derivation

**Figure 2:**
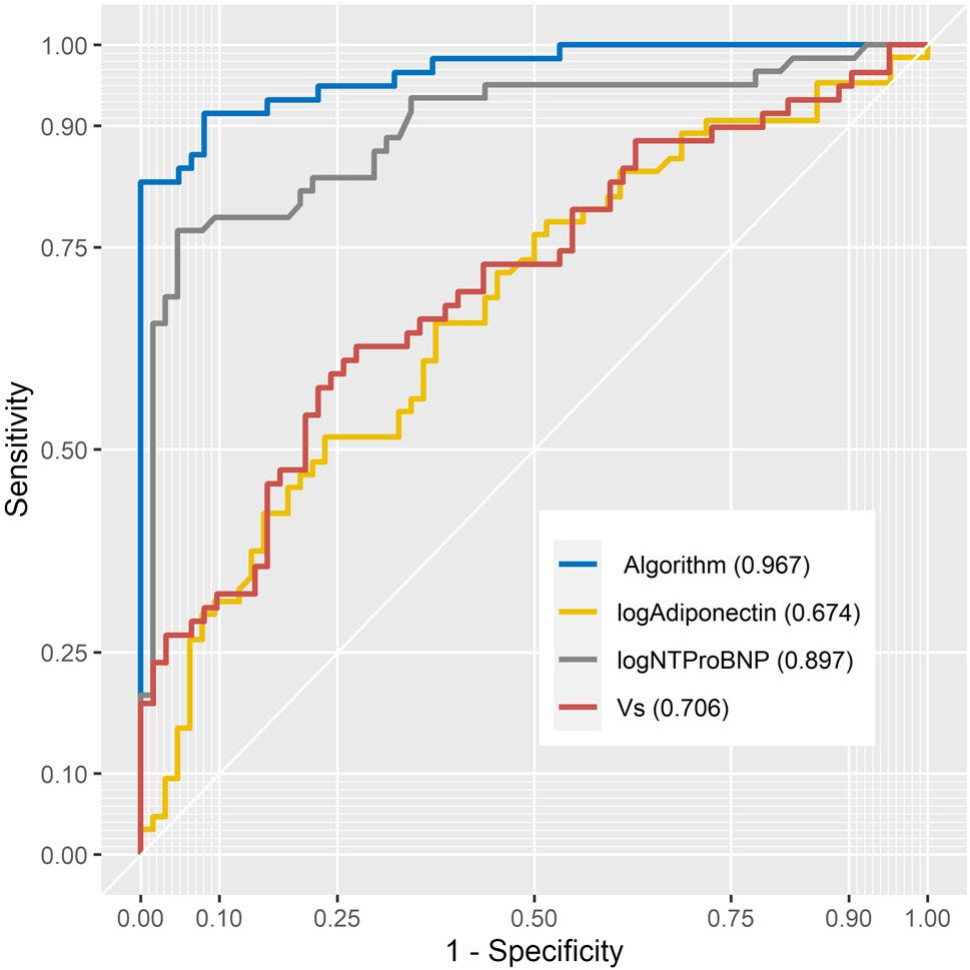
ROC curve of ocular-biochemical score.

Combining biomarker and ocular measurements may provide a novel method of identifying individuals at risk of CAD. Using forward and backward Wald logistic regression identified the combination of Vs, NT-proBNP and adiponectin as having the highest predictive ability to discriminate the post-MI group from control; with the fewest variables suitable for the patient cohort size for the study (χ^2^(3) = 111.74, p < 0.05). The model explained 80.4% (Nagelkerke *R*^2^) of the variance in CAD and correctly classified 90.1% of cases. Hosmer and Lemeshow test for goodness of fit was χ^2^(8) = 6.83, p = 0.55; sensitivity 93.0%, specificity 91.5%, positive predictive value (PPV) 91.4%, negative predictive value (NPV) 93.1%; area under receiver operator characteristic curve (AUROC) 0.967 (Table 4 and Fig. 2). In comparison, the QRISK3 score had a reduced sensitivity (71.7%), specificity (64.9%), PPV = 68.3% and NPV = 68.5% as shown in Table 4 and Supplementary Fig. 1.

**Table 4:** Multivariate logistic regression with biomarkers and ocular parameters for detecting CAD.

Biomarkers/ocular parameters	AUROC	Sensitivity %	Specificity %	PPV%	NPV%
Log_10_ NT-proBNP, adiponectin, log_10_ H-FABP, Va	0.977	94.7	91.5	91.5	94.7
Log_10_ NT-proBNP, log_10_ adiponectin, log_10_ H-FABP, Vs	0.976	93.0	93.2	93.0	93.2
**Log**_**10**_** NT-proBNP, log**_**10**_** adiponectin, Vs**	**0.967**	**93.0**	**91.5**	**91.4**	**93.1**
Log_10_ NT-proBNP, log_10_ adiponectin, Va	0.966	94.7	93.2	93.1	94.8
Log_10_ H-FABP, log_10_ NT-proBNP, Va	0.953	88.1	91.9	91.2	89.1
Log_10_ H-FABP, log_10_ NT-proBNP, Vs	0.953	86.4	90.3	89.5	87.5
Log_10_ H-FABP, log_10_ NT-proBNP, log_10_ WSR	0.936	84.7	88.7	87.7	85.9
Log_10_ NT-proBNP, Vs	0.926	85.0	83.9	83.6	85.2
Log_10_ NT-proBNP, Va	0.925	83.3	85.5	84.7	84.1
IL-6, Vs	0.840	76.2	76.2	76.2	76.2
Log_10_ H-FABP, Vs	0.830	73.0	76.6	75.4	74.2
Log_10_ H-FABP, Va	0.815	73.0	71.9	71.9	73.0
CRP, D	0.810	79.4	67.2	71.4	75.9
Log_10_ adiponectin, log_10_ WSR	0.769	76.3	60.3	66.2	71.4
Log_10_ adiponectin, Va	0.721	76.2	53.1	61.5	69.4
Log_10_ adiponectin, D	0.702	72.1	54.2	62.0	65.3
Vs	0.706	63.1	73.4	70.7	66.2
Log_10_ adiponectin	0.674	65.6	62.5	63.6	64.5
Log_10_ NT-proBNP	0.897	78.7	90.6	88.9	81.7
QRISK3 score	0.732	71.7	64.9	68.3	68.5
Age (years)	0.604	59.1	57.6	58.2	58.5

Significant values are in bold.

*CRP* C-reactive protein, *H-FABP* heart-type fatty acid-binding protein, *IL-6* interleukin-6, *NT-proBNP* N-terminal pro brain natriuretic peptide, *D* vessel diameter, *Va* axial velocity, *Vs* cross-sectional velocity, *WSR* wall shear rate, *AUROC* area under receiver operator characteristic curve, *PPV* positive predictive value, *NPV* negative predictive value.

## Discussion

The aim of this case–control study was to evaluate the feasibility for using conjunctival microvascular parameters assessed non-invasively, in combination with biomarkers as a potential screening modality for CAD. This was achieved by comparing conjunctival imaging parameters and blood-based biomarkers in patients with established CAD and severe CVD phenotype (post-MI) with a control group of healthy volunteers.

Of the five conjunctival microvascular parameters assessed, four (diameter, axial velocity, cross-sectional velocity and wall shear rate) were significantly different between the post-MI and controls. Conjunctival vessel diameter was significantly higher in the post-MI group, however axial and cross-sectional velocities were lower compared with the control group. In addition, wall shear rate was lower in the post-MI group. An algorithm which included both conjunctival and biomarker measurements (microvascular velocity, adiponectin and NT-proBNP) was identified that differentiated the post-MI from control (AUROC 0.967, sensitivity 93.0%, specificity 91.5%) (Table 4 and Fig. 2). Application of this novel algorithm could be used to screen asymptomatic individuals for atherosclerotic disease.

Reduced blood velocity, elevated NT-proBNP and lower adiponectin reflect pathological cardiovascular conditions. The main function of the microcirculation is regulation of vascular resistance and oxygen perfusion of tissues. Therefore, decreased velocity and increased vessel diameter in the post-MI group, opposes the normal Poiseuille's law of blood flow dynamics, suggesting underlying microvascular dysfunction^42^.

Adiponectin and NT-proBNP are implicated in the pathogenesis and pathophysiology of CAD, for example, low levels of adiponectin (< 4.0 µg/mL) have been reported to be associated with an increased risk of CAD^43^. Low levels of adiponectin reflect underlying abnormal lipid metabolism, inflammation and is a contributor of atherosclerosis and subsequent vascular events^43–45^.

This study evaluated biomarkers of inflammation and endothelial dysfunction associated with CAD. A reduced conjunctival wall shear rate was observed in post-MI compared with control. Potential screening algorithms for CAD incorporating biomarkers and conjunctival blood vessel parameters have been identified (Table 4). These data suggest that changes in ocular microvascular parameters e.g. cross-sectional velocity, combined with biomarkers, have potential benefits for detecting atherosclerotic heart disease.

Detecting inflammation and endothelial dysfunction may offer the earliest opportunity to institute primary preventative therapies, lowering the risk of major adverse cardiac events. Endothelial dysfunction occurs in the initial stages of the ischemic cascade by promoting the development of atherosclerosis^46^. Reduction in wall shear rate and stress are associated with atherosclerotic plaque development and progression in coronary microvascular dysfunction^42,47^. Endothelial cells are regulators of inflammation; chronic inflammation is also an etiology of endothelial dysfunction. Hence, an understanding of the relationship and underlying pathophysiological mechanisms of endothelial dysfunction and inflammation is vital.

The authors of this report acknowledge that MI triggers inflammation aimed at enhancing healing and tissue repair which resolves between 2 and 4 weeks post-infarction^48–50^. Considering the recruitment interval of the post-MI group, post-infarct inflammation potentially could contribute to the inflammatory mediators released to promote healing^51^. However, the mediators have the potential of aggravating the existing atherosclerotic plaques leading to recurrent MI. Assessment of the relationship between cross-sectional velocity and biomarkers of endothelial dysfunction has potential utility in the screening of both asymptomatic and symptomatic CVD for stratifying risk of future events. This report suggests that the proposed algorithm identified in this study is tested and validated in a follow up study.

## Study limitations

The small sample size had a tendency of generating false-positive errors (type 1)^52^. Hemorheological parameters respond differently to the cardiac cycle^53^. Hence, lack of differentiation of vessels has the potential of concealing true parameter values leading to biases in results and conclusions. Another study limitation relates to known confounding factors of vascular alteration including, but not limited to, age, lifestyle and medical factors^5^. Underlying medical factors with known effects on microvasculature include systemic blood pressure, hypercholesterolaemia, and diabetes. Heart failure and lifestyle factors such as smoking have known effects on the microvasculature. We acknowledge the sample size as the major study limitation on statistics on confounding factors, comorbidities, routine medication and hence recommend a larger study population for validation.

## Conclusion

This study demonstrated the potential for a non-invasive ocular screening modality to detect conjunctival microvascular dysfunction in patients with overt atherosclerotic heart disease. The novel algorithm identified, combines ocular parameters with biomarkers that could distinguish between control and post-MI groups. The assessment of the conjunctival microcirculation is a rapid non-invasive test that can easily compliment blood investigations and existing lifestyle factors for CVD screening and risk stratification; however, this would need further validation as a screening tool in a larger sample of asymptomatic people.

## Supplementary Information

Supplementary Figure S1.
