# Supplementary material for: A novel algorithm for cardiovascular screening using conjunctival microcirculatory parameters and blood biomarkers
Source: Sci Rep. 2022 Apr 21;12:6545. doi: 10.1038/s41598-022-10491-7 (PMC9023476; doi:10.1038/s41598-022-10491-7)
Supplement: Supplementary file 1 — Supplementary Figure S1. [file 41598_2022_10491_MOESM1_ESM.pdf]

A novel algorithm for cardiovascular screening using conjunctival microcirculatory parameters and blood biomarkers

Author list

Agnes Awuah[1], Julie S. Moore[1], M. Andrew Nesbit[1], Mark Ruddock[2], Paul F. Brennan[3], Jonathan A. Mailey[3], Andrew J. McNeil[4], Min Jing[5], Dewar D. Finlay[5], Emanuele Trucco[4], Mary Jo Kurth[2], Joanne Watt[2], John Lamont[2], Peter Fitzgerald[2], Mark S. Spence[3], James A. D McLaughlin[5], Tara C.B. Moore[1]*

Corresponding author: *Professor Tara Moore (tara.moore@ulster.ac.uk)

Institutions

[1]Biomedical Sciences Research Institute, Ulster University, Cromore Road, Coleraine, BT52 1SA, UK

[2]Randox Laboratories Ltd, Clinical Studies Group, Antrim BT29 4QY, UK

[3]Department of Cardiology, Royal Victoria Hospital, Belfast Health and Social Care Trust, 274 Grosvenor Road, Belfast BT12 6BA, UK

[4]VAMPIRE project, Computing (SSEN), University of Dundee, Dundee DD1 4HN, UK

[5]Nanotechnology and Integrated Bioengineering Centre (NIBEC), Ulster University, Jordanstown BT37 0QB, UK

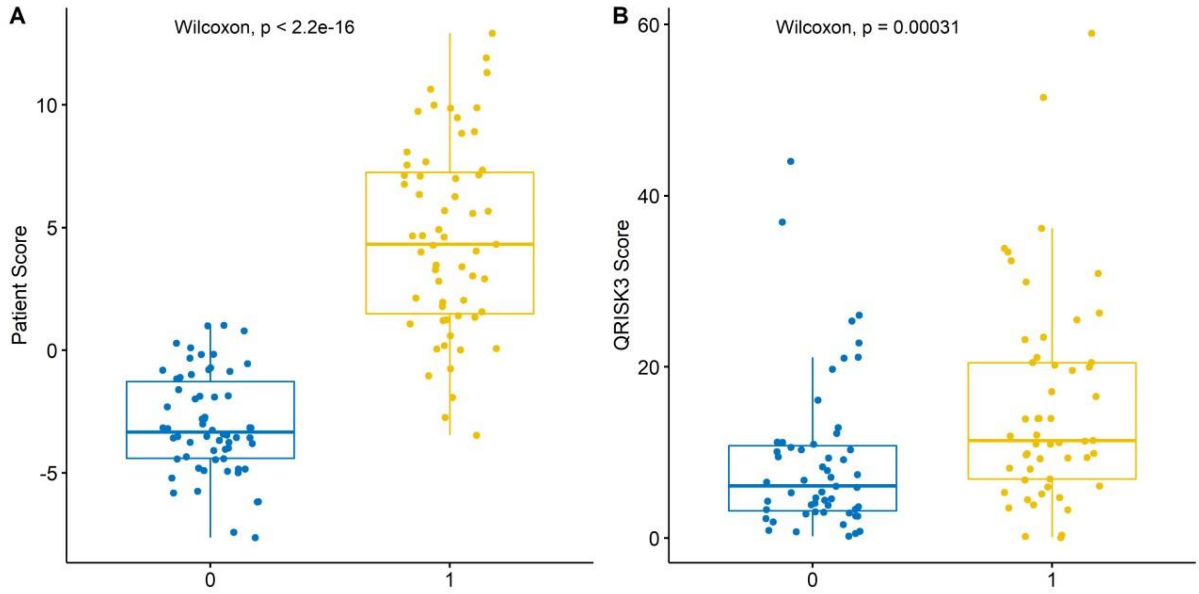

Supplementary Figure 1: Box plots comparing Patient Score to the QRISK3 score

*The Patient Score was derived from our algorithm. The linear regression equation for Patient Score = 7.22 + -30.211*Vs + -3.248*log*$_{10}$*adiponectin + 2.391*log*$_{10}$*Nt-proBNP.*
